# A Case Report of Hypophosphatemia Leading to the Diagnosis of Mesothelioma

**DOI:** 10.7759/cureus.25285

**Published:** 2022-05-24

**Authors:** Zi W Goh, Faisal Hasan

**Affiliations:** 1 Diabetes and Endocrinology, Hastings Hospital, Hastings, NZL; 2 Diabetes and Endocrinology, Royal United Hospital, Bath, GBR

**Keywords:** phosphate, malignant mesothelioma, fibroblast growth factor-23, hypophosphatemia, case report

## Abstract

Hypophosphatemia can be commonly encountered as an electrolyte imbalance and is defined as a value less than 0.8 mmol/l (2.5 mg/dl). It can be an incidental finding, but it is not uncommon to see it presenting with varied symptoms. It is good to have a clear diagnostic approach to this so adequate treatment can be instated.

We present a 66-year-old gentleman who presented with hypophosphatemia. Investigations confirmed renal phosphate wasting secondary to fibroblast growth factor-23 (FGF-23). Imaging showed right pleural effusion, and pleural biopsy confirmed malignant mesothelioma. This may just be an association rather than the cause of his hypophosphatemia. It does however highlight the importance of further investigations for patients with tumor-induced osteomalacia.

## Introduction

Hypophosphatemia can be a common electrolyte problem in the acute hospital setting, but it can also be seen in outpatient clinics. The symptoms of hypophosphatemia are varied depending on the level and rate of depletion and are non-specific, therefore the diagnosis is usually based on biochemical findings [1].

The causes are also numerous, and it is useful for the clinician to have a diagnostic road map on how to investigate this. In this case report, we describe an unusual diagnosis of malignant mesothelioma resulting from the investigations for hypophosphatemia. We also suggest a pragmatic method for the diagnostic workup of hypophosphatemia, which can be used in a busy clinical setting.

## Case presentation

A 66-year-old man, a retired office worker, was referred with a two-year history of lethargy, cramps in his hands, and generalized bony aches and pains. He did not have any history of confusion, muscle weakness, or seizures. His weight was stable, and he had a good appetite. On systems review, he had no other symptoms. His past medical history included myocardial infarction at the age of 58 years, glaucoma, and benign prostatic hypertrophy. His regular medications included aspirin, cetirizine, ramipril, tamsulosin, and finasteride. There was no significant family history of note. Socially, he was a never-smoker and drank 14 standard units of alcohol per week.

On initial investigation, his phosphate was 0.5 mmol/L (0.8 - 1.5), which was confirmed to be low on repeat testing on two other occasions. His magnesium was normal at 0.9 mmol/L (0.7 - 1.0) and calcium was 2.3 mmol/L (2.2-2.6). Other biochemical investigations showed parathyroid hormone (PTH) 3.9 pmol/L (1.6-6.9), vitamin D 79 nmol/L (>50), coeliac screen negative, thyroid-stimulating hormone (TSH) 3.42 mIU/L (0.3-5.0), creatinine 91 umol/L, and estimated glomerular filtration rate (eGFR) 75 ml/min/1.73 m^2^ (Table 1).

**Table 1:** Initial biochemical results for the case eGFR: estimated glomerular filtration rate

Test (unit)	Patient	Reference range
Phosphate (mmol/L)	0.5	0.8-1.5
Magnesium (mmol/L)	0.9	0.7-1.0
Calcium (mmol/)	2.3	2-2.2.6
Parathyroid hormone (pmol/L)	3.9	1.6-6.9
Vitamin D (nmol/L)	79	>50
Creatinine (umol/L)	91	
eGFR (ml/min/1.73 m^2^)	75	
24-hour urine phosphate excretion (mmol/24 hour)	20.2	13.0-42.0
1,25 dihydroxyvitamin D (pmol/L)	87	55 -139
Fibroblast growth factor (RU/ml)	101	<100

He had a short course of phosphate supplementation, which improved some of his symptoms although he had ongoing symptoms of lethargy and feeling that his muscles were weaker than usual.

A 24-hour urine collection was performed to look for renal phosphate wasting. This showed urine volume of 2.68 L, phosphate 7.55 mmol/L, and phosphate excretion of 20.2 mmol/24 hours (13.0-42.0), which confirmed urinary phosphate loss.

Further biochemical investigations were performed, including 1,25 dihydroxy (OH)2 vitamin D 87 pmol/L (55-139) and fibroblast growth factor-23 (FGF-23) 101 RU/ml (<100). This confirmed FGF-23-mediated hypophosphatemia.

**Figure 1:**
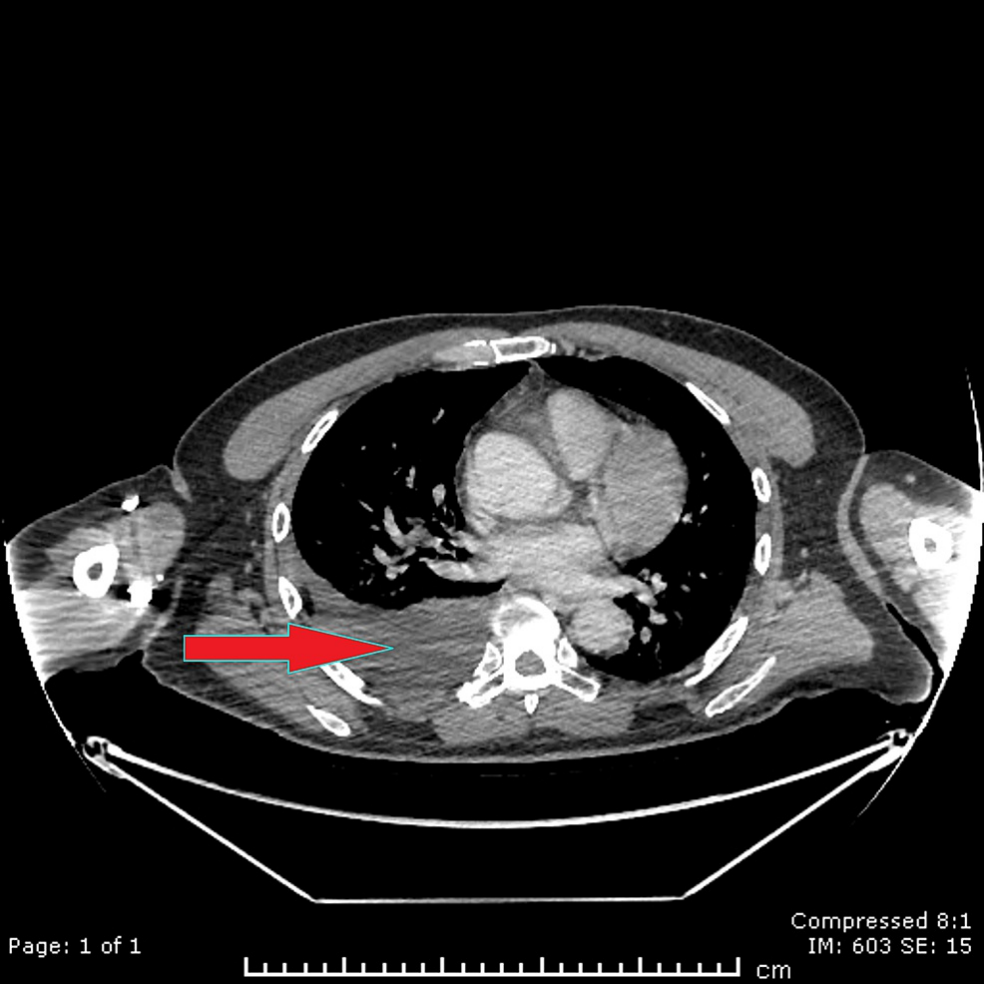
Right-sided pleural effusion clearly seen on axial imaging Computerized tomography of the chest

Imaging was performed to look for the source of FGF-23. A fluorodeoxyglucose-positron emission tomography (FDG-PET) scan showed incidental left adrenal nodule and large right pleural effusion (Figure 1). Investigations performed for the incidental left adrenal nodule showed this to be a non-functioning adenoma.

The right pleural aspirate showed clusters of atypical mesothelioma cells, suggestive of mesothelioma. Right parietal pleural biopsy via video-assisted thoracoscopic surgery (VATS) confirmed malignant mesothelioma. He went into a trial for the treatment of mesothelioma and had pleurectomy decortication and adjuvant chemotherapy with cisplatin and pemetrexed. His phosphate has normalized without the use of supplements.

## Discussion

There is no known prevalence or incidence of hypophosphatemia in the general population. The symptoms of hypophosphatemia are varied and can affect multiple organ systems. Symptoms of chronic hypophosphatemia can include muscle weakness, bone pain, and arthritis [1]. This patient had non-specific muscular symptoms, and the diagnosis of hypophosphatemia was confirmed on routine biochemical testing.

Phosphate homeostasis occurs from a balance between urinary phosphate losses, net phosphate absorption from the gastrointestinal tract, and equal amounts deposited and reabsorbed from bone [2]. There are multiple endocrine feedback loops involving PTH, FGF-23, and 1,25 (OH)2 vitamin D that control the phosphate balance [3]. The normal renal response to low serum phosphate is to increase phosphate reabsorption, leading to minimal phosphate excretion in the urine [4]. Most of the filtered phosphate is reabsorbed in the proximal tubule via the sodium-phosphate cotransporters [4].

The main mechanisms that can explain a decrease in serum phosphate are (1) internal redistribution, i.e. from extracellular to intracellular; (2) decreased gastrointestinal phosphate absorption; and (3) excessive urinary phosphate loss [3].

We would like to set out an approach for patients with hypophosphatemia. It is important to measure serum phosphate on a morning fasting blood specimen, as serum phosphate can vary with food intake [1]. Once hypophosphatemia is confirmed, the next step is to rule out any acute clinical condition that can cause internal redistribution of phosphate, for example, acute respiratory alkalosis, refeeding syndrome, or diabetic ketoacidosis [2].

It is also essential to obtain a complete pharmacological history to identify the use of drugs that can be associated with hypophosphatemia such as intravenous iron, phosphate-binding antacids, and anti-retroviral drugs [1]. In addition, evaluation should include personal and family history to determine the age of onset of symptoms and the presence of other associated abnormalities such as dental problems [1]. In general, the older the patient, the more likely it is to be an acquired disorder.

The next step is to establish the presence of renal phosphate wasting. There are several ways of doing this. One paper has recommended that 24-hour urine phosphate excretion above 100 mg (3.2 mmol) is indicative of renal phosphate wasting in patients with hypophosphatemia [5]. Other papers have suggested calculating the tubular reabsorption of phosphate (TRP) or the renal tubular maximum reabsorption of phosphate per liter of glomerular filtration rate (TmP:GFR) [1]. TRP and TmP:GFR calculations require simultaneous urine and serum phosphate and creatinine measurements. Vitamin D deficiency must be corrected and phosphate supplementation should be stopped prior to the tests. Renal phosphate wasting is confirmed with either low TmP:GFR or TRP < 85-95% [1].

If renal phosphate wasting is confirmed, serum calcium, 1,25 (OH)2 vitamin D, PTH, and FGF-23 should be measured [1]. The typical biochemical pattern of FGF-23-mediated diseases is characterized by normal serum calcium and PTH, low or low-normal 1,25 (OH)2 vitamin D, and inappropriately normal or high FGF-23.

Tumor-induced osteomalacia is an acquired cause of FGF-23-mediated hypophosphatemia and is usually caused by phosphaturic mesenchymal tumors [6]. Usually, to locate the phosphaturic mesenchymal tumors, functional imaging studies, such as 68Ga DOTATATE PET/CT, should be used first, followed by anatomical imaging with CT or MRI [7]. There are case reports of metastatic malignant tumors, such as small cell carcinomas, which can also have FGF-23-mediated hypophosphatemia [8]. It is possible that the diagnosis of mesothelioma in our patient is a casual association rather than the cause of the hypophosphatemia. Surgical resection is the treatment of choice for tumor-induced osteomalacia.

## Conclusions

This case report described a case of mesothelioma diagnosed after having investigations for hypophosphatemia. This may be coincidental rather than the causative factor of hypophosphatemia. A clear diagnostic approach may help in arriving at a diagnosis sooner. It is important to remember to test the blood phosphate level on a fasting sample. A detailed pharmacological history will prevent unnecessary investigations if the cause is found in the medications being taken by the patient. We also highlight the importance of investigations for increased urinary loss causing hypophosphatemia; otherwise, it would be possible to miss a serious diagnosis in patients such as tumor-induced osteomalacia. 
